# Capability of Integrated MODIS Imagery and ALOS for Oil Palm, Rubber and Forest Areas Mapping in Tropical Forest Regions

**DOI:** 10.3390/s140508259

**Published:** 2014-05-07

**Authors:** Sheriza Mohd Razali, Arnaldo Marin, Ahmad Ainuddin Nuruddin, Helmi Zulhaidi Mohd Shafri, Hazandy Abdul Hamid

**Affiliations:** 1 Institute of Tropical Forestry and Forest Products, Universiti Putra Malaysia, Serdang 43400, Selangor, Malaysia; E-Mails: ainuddin@upm.edu.my (A.A.N.); hazandy@gmail.com (H.A.H.); 2 Departamento de Ecología e Hidrología, Universidad de Murcia, Murcia 30100, Spain; E-Mail: arnaldo@um.es; 3 Faculty of Engineering, Universiti Putra Malaysia, Serdang 43400, Selangor, Malaysia; E-Mail: helmi@upm.edu.my

**Keywords:** accuracy mapping, forest, oil palm, rubber, tropical regions, ALOS, MODIS

## Abstract

Various classification methods have been applied for low resolution of the entire Earth's surface from recorded satellite images, but insufficient study has determined which method, for which satellite data, is economically viable for tropical forest land use mapping. This study employed Iterative Self Organizing Data Analysis Techniques (ISODATA) and K-Means classification techniques to classified Moderate Resolution Imaging Spectroradiometer (MODIS) Surface Reflectance satellite image into forests, oil palm groves, rubber plantations, mixed horticulture, mixed oil palm and rubber and mixed forest and rubber. Even though frequent cloud cover has been a challenge for mapping tropical forests, our MODIS land use classification map found that 2008 ISODATA-1 performed well with overall accuracy of 94%, with the highest Producer's Accuracy of Forest with 86%, and were consistent with MODIS Land Cover 2008 (MOD12Q1), respectively. The MODIS land use classification was able to distinguish young oil palm groves from open areas, rubber and mature oil palm plantations, on the Advanced Land Observing Satellite (ALOS) map, whereas rubber was more easily distinguished from an open area than from mixed rubber and forest. This study provides insight on the potential for integrating regional databases and temporal MODIS data, in order to map land use in tropical forest regions.

## 1. Introduction

The natural land cover of the Peninsula of Malaysia is primarily evergreen forests, including mountain, hill, and lowland tropical forests, along with peat swamps and mangrove forests in the lake and river regions. The most significant land use change in the peninsula has been the clearing of forests for agricultural purposes and mining activities, as well as for the establishment of settlements along the coastal and riverine areas [1]. The conversion of natural forest into agricultural uses such as for oil palm, rubber, coconut, pineapple, mixed horticulture, market gardening and floral farms, has been reflected in regional land use maps of the peninsula. By the 1960s, the Malaysian Agricultural Department had successfully produced the first land use classification maps for the West of Malaysia, with the cooperation of the Canadian Government. To date, the maps have been updated every two years based on soil surveys, satellite image interpretation, digitizing and ground verification through the utilization of satellite imagery such as aerial photos, Landsat Thematic Mapper (TM) and System Probatoire d'Observation de la Terre (SPOT). Utilization of high resolution satellite such as SPOT proved reputable in previous studies, since satellite imagery has been used for decades in many areas such as evergreen tropical forest and riparian studies [2,3]. The process, however, is very expensive, requiring extensive human labour to interpret the results, maintain the software and monitor the equipment [4]. Consequently, although land use maps for Peninsular Malaysia are available in digital format to the related government agencies, private or non-governmental sectors, non-profit making nature society, environmental public researchers and scientists have not been able to acquire these data because of the high cost.

The classification of satellite imagery for land cover mapping requires the extensive skills of an experienced analyst [5]. When such skills were not available, land cover classification maps have been developed through ground surveys and base maps such as digital topographic maps, recent land use maps and soil suitability agricultural maps; these techniques have been increasing the accuracy of land cover classification maps [6]. Updating or replacing these maps with a large amount of remotely sensed data remains a very challenging task [7]. Yet both the private sector, governmental and non-governmental agencies are now depending on satellite applications for mapping their land uses. For example, the United States Geological Survey's Gap Analysis Program, which started in 1998 [8], and the National Land Use Change Program of China [3], rely on such data.

The 10th Conference of the Parties for the Convention on Biological Diversity, held in Japan, was aimed at achieving the Aichi Biodiversity Targets, whose goal is to at least halve and, where feasible, bring close to zero, the rate of loss of natural habitats, including forests, and to establish a conservation target of 17% of terrestrial and inland water areas and 10% of marine and coastal areas. One of the most crucial sectors where Earth Observation (EO) can assist in such land use and land cover mapping is by enabling the mapping of large inaccessible areas. Hence EO is playing a major role in providing essential tools to support national and international monitoring systems [9]. The objective of this study was to provide techniques for mapping land uses such as evergreen forests, oil palm and rubber farming, and other land use types.

**Figure 1.**
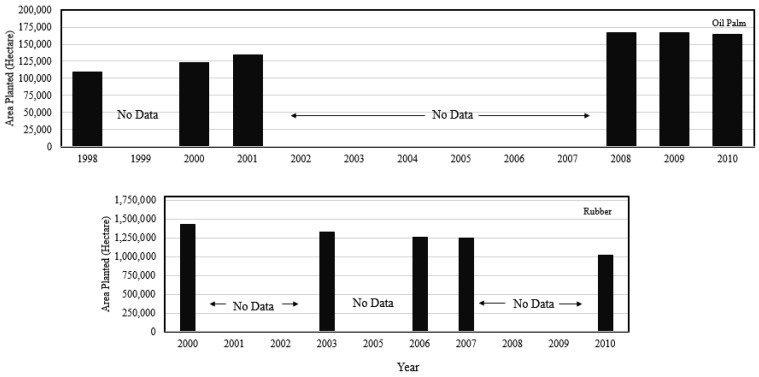
Graphs of area planted with oil palm and rubber from 1998 to 2010.

The rubber industry, in particular, is being given special attention, as it has great economic potential and provides income for over 400,000 small landholders. The area planted in oil palm has expanded year by year. In 1998 it was planted with 109,446 ha; this was increased to 123,343 ha in 2000 and to 134,427 ha in 2001. The area reached a maximum of 171,647 ha in 2008 but was reduced to 166,501 ha in the next year, and has continued to fall, to 164,362 ha in 2010 [10]. The rubber plantation scenario presents a different pattern, as reported by the report. Rubber was planted in 1,430,680 ha in 2000, 1,325,600 ha in 2003, and 1,263,590 ha in 2006, and consistently dropped from 2007 to 2010 (1,248,040 to 1,020,380 ha) (Figure 1). In addition, the National Key Economic Area (NKEAs) of Malaysia report identified oil palm and rubber as priority areas for contributing most of Malaysia's economic performance by 2020 [11].

With the increasing global demand for oil palms (at least before 2008) and rubber products, it is necessary to develop and update land use maps for improving our understanding of land use changes, with minimal labour and equipment cost. Furthermore, such maps provide information not only on existing land use types such as tropical evergreen forests, oil palm and rubber, but also on other agricultural uses such as pineapple, cocoa, mixed horticulture and other crops.

## 2. Material and Methods

### 2.1. Study Area

**Figure 2.**
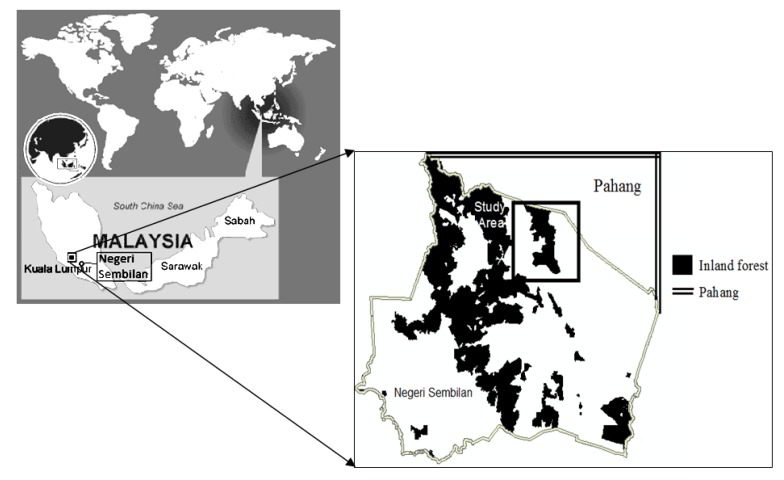
Map of the study area showing the study area at lowland dipterocarp forest of inland forest and state of Pahang bordering the study area.

Negeri Sembilan is located in the western part of the Peninsula of Malaysia. Research was conducted in an area of slightly more than 1,000 km^2^ centered around the Pasoh Forest Reserve (PFR). The PFR is located at 2°58′N, 102°18′E (Figure 2). It is connected to urban areas by the Kajang-Seremban Highway (E21), road number 86 and N23; travel time is about 2 hours and 15 minutes from the Federal Territory of Kuala Lumpur. The PFR is covered with primary lowland mixed dipterocarp forest (tropical evergreen broadleaf forest) that includes various species of Shorea and Dipterocarps [12]. There are numerous types of vegetation in the area surrounding the forest reserve. The oil palm plantations of Felda Pasoh Dua (PFR Corridor) are dominant, covering the southern region and Felda Pasoh Empat in the northern part of the area. At the other site of the PFR is Felda Lui Barat, which is planted with both oil palm and rubber. Mean temperature recorded is 26.3 °C measured for 2002–2005. Recent annual precipitation is 1,702 mm measured for 2000–2011 [13–15]. Historically, most of the surrounding area has been natural forests, but human exploitation has led to a significant decrease in these primary forests, as they are turned into oil palm plantations [16], with a total area of 568,561 ha planted in the peninsula by 1975 and dramatically increased for more than 1 million ha by 20 years.

The objective of this study was to provide techniques for mapping land uses such as evergreen forests, oil palm and rubber farming, and other land use types. Negeri Sembilan, the location for the permanent research plot of Pasoh Forest Reserve (PFR) in Southeast Asia, was chosen as the central point of the study area. The plot was used for intensive biomass and productivity research from 1971–1973, under the International Biological Programme (IBP), Universiti Malaya (UM) and the UNESCO Biosphere Program (MAB), and the joint Rainforest Research Project of Universiti Malaya and the University of Aberdeen, UK. The surrounding PFR is representative of the dramatic changes in land use and land cover during the past few decades in Negeri Sembilan. The Negeri Sembilan region is a critical area for both oil palm and rubber production, and was chosen as a focus of the Malaysia Government's Economic Transfer Programme.

### 2.2. MODIS Data, Pre-Processing and Enhancement

To carry out the objectives of this study, MODIS Surface Reflectance series data (MOD09A1) acquired in 2000, 2005 and 2008 were used. MODIS Land Cover products (MOD12Q1) was taken in 2001, 2005 and 2008 and ALOS was taken in 2008 (Table 1). The 500 m MOD09A1 series data of 2000, 2005 and 2008, which could potentially be used for land use mapping [17], was been inter-calibrated with other data such as National Oceanic and Atmospheric Administration Advanced Very High Resolution Radiometer (NOAA AVHRR) and linked to field census data such as in [18].

Images were selected based on scale, availability of the image data, cost, time constraint and atmospheric correction [20]. The MOD09A1 500 m resolution was chosen because it's covers the whole study area with one scene, hence, reducing times and cost for mosaicking the imageries. The images were collected based on the availability of the image with minimum cloud cover, which could decrease precision during image interpretation and classification. The high temporal resolution promotes good quality imagery with limited cloud contamination [21]. Unfortunately, good quality satellite data is often particularly difficult to obtain in tropical forest areas due to lower seasonality and heavy cloud cover conditions [22]. We have downloaded more than fifty images for those years and reanalysed them with band matching for filtering a high quality image. Finally, with these disadvantages only one individual image were identified for each year for further processed. Higher frequencies of bright pixels were detected on forested areas, because clouds are generally bright in the visible spectrum and cold in the infrared spectrum. Therefore, to overcome these disadvantages cloud removal analysis were conducted using density slice and masking procedure techniques in Exelis Visual Information Solution (ENVI). In this study, cloud detection procedure were conducted based on comparison with Present Land Use map of Negeri Sembilan 2004 and the images in visible and infrared bands (focusing in band 1, band 2 and band 6), where cloud cover is the unwanted information in optical images. Furthermore, image enhancement were conducted using band combination techniques of: (i) 6, 4, 3; (ii) 1, 2, 3; (iii) 1, 3, 4; (iv) 5, 3, 4; (v) 3, 1, 2; and (vi) 2, 6, 1. The images were also enhanced using histogram equalization for further image interpretation [23]. MOD09A1 of 2000 and 2005 image were validated with Present Land Use map of Negeri Sembilan 2004. The land use map is updated every two years and reproduced with recent SPOT image and JUPEM (Malaysian Survey and Mapping Department), Topography Map Series 7030, which further verified with ground survey by land surveyor [24]. First, the map was geo-corrected using Topography Map Seremban 1996 Series 7030 and resample to 500 m pixel sizes as the same size of MOD09A1 data. The map was subset into an area of interest by using an areas similar with MOD09A1 data.

MOD12Q1 500 m resolution was chosen based on availability of the image that was first produced from 2001. Therefore, we chose MOD12Q1 2001 data to compare with our land use classification from MOD09A1 2000 data. The MOD12Q1 2005 and 2008 were fortunately available for our study. ALOS had to order from our satellite data vendor, Satellite Imaging Corporation (SIC), therefore much time consuming waiting for choosing the recent data, suitable image with minimal cloud cover, acquiring, pre-processing and mapping. We found ALOS 2008 was the best image data available for the study area.

The study was conducted in four parts: (1) creating a MOD09A1 500 m land use classification map, employing unsupervised ISODATA and K-Means classification techniques; (2) creating an ALOS 10 m land cover types map from reclassification, proximity analysis and spatial analyst; (3) creating an elevation map from NFI-4 data; and (4) comparing the MODIS land use classification map with ground verification survey, NFI-4 data, Topographic data 1997, MOD12Q1, ALOS land cover type and elevation.

### 2.3. ALOS AVNIR-2 Data and Processing

The ALOS is ALOS AVNIR-2 or Advanced Land Observing Satellite of Advanced Visible and Near Infrared Radiometer type 2 with 10 m resolution. The ALOS 2008 image was enhanced utilizing histogram equalization that was found to be effective at improving image interpretation for land uses such as rubber, oil palm plantations and forested areas [23].

### 2.4. Image Classification and Unsupervised Classification (ISODATA and K-Means)

Although many computer-aided techniques have been developed for land cover classification, the skills and experience of an analyst are still very important to the success of the image classification [5,20]. We chose ISODATA because our study area consisted a less complex land cover types, consisting forested areas and agricultural plantation mostly an oil palm or rubber—which are widespread in the peninsula. ISODATA is a suitable technique to be applied in forested areas with presence of agricultural plantations because most of forested areas which have been previously logged several years ago may have excellent ancillary data. Data such as land-use maps, national land cover maps and as well as a good local knowledge of the terrain, vegetation and soil of an area are essential databases for logging managers. Therefore, the data is possibly to be acquired and employed in ISODATA classification for this area or other similar background area.

K-Means was chosen because the study area consisted with forested areas within lowland and hilly dipterocarp and also non-dipterocarp, peat swamp and mangrove forest. Most of forest and land managers in tropical forest were updated with new technology of land mapping. This is because they should facilitate ecological and monitoring systems with the aim of providing useful guidance on forest information included forests dynamics, regeneration, *etc.* [25]. Therefore, with this current situation most of the information databases required for the classification are highly available. Because the K-means clustering technique is simple, where K is the desired number of clusters to be input, highly available database number increased the number of K. The classification adopted in this study is therefore applicable to the background of the study area. We therefore chose to adopt unsupervised classification, to overcome the challenges of mapping land use in a tropical region using low-resolution satellite imagery.

### 2.5. Mapping Land Use Classification

#### 2.5.1. Mapping MODIS

The initial observations were conducted on a topographic map of Seremban and Kuala Pilah 1997; and Present Land Use maps of Negeri Sembilan from 1997 and 2004 as a base map for the classification. The land use map was produced by the Malaysian Agricultural Department whose study found that the land use map was a good background to present the land use classification map for the 2000–2008 MODIS data set, since there had been no conversions of forest land to oil palm plantations at the border of PFR since 1997. The maps were registered using Rectified Skewed Orthomorgraphic (RSO) coordinate format, the format that has been utilized by Malaysian government agencies such as the Malaysian Forestry Department in registering their map for further image processing, analysis, spatial applications and also for decision making (*i.e.*, forest fire risk assessment and forest resource updating). Furthermore, the maps were rectified based on Nearest Neighbor, 1st Order Polynomial with pixel size of 500 m and were projected to WGS 84, UTM Zone 48 N. Geo-correction was based on four points: (i) an area at the boundary of Negeri Sembilan/Pahang; (ii) an area bordering the oil palm plantation and PFR, of which the nearest point indicated in Google Earth is Kampung Lui; (iii) PFR, which is the nearest point to Felda Pasoh Dua; and (iv) PFR and an area bordering a rubber plantation in the southern part of PFR; in this study we used Google Earth images to locate points for image registration for this point [4]

Unsupervised classification of ISODATA Gamma (ISODATA-1), ISODATA Kuan (ISODATA-2), K-Means Gamma (K-Means-1) and K-Means Kuan (K-Means-2) were employed in the study area as depicted in Table 2. The ISODATA was determined using maximum likelihood decision rule to calculate class mean that are evenly distributed in the data space and then iteratively clusters the remaining pixels, using minimum distance techniques [26,27]. The K-Means was determined by following the methodology found in [28], which the classification was conducted using the Erdas Imagine 9.1 software. Parameters incorporated in the analysis for ISODATA were reported as the following: number of classes at minimum 5 and maximum 10; minimum pixel in classes, 1; minimum class distance, 5; and minimum merge pairs, 2. Finally, the clusters were classified in terms of the ground conditions they represented, identified from the ground survey and land-use maps of 1997 and 2004 [29]. The parameter for K-Means arranged was the number of classes at minimum 5. The Gamma and Kuan applied in the study following the methodology from [30], tested for pixels filtering at 3 × 3 and 5 × 5 pixels window. After preliminary classification, 5 × 5 pixels window classification were highlighted and applied to all the images.

#### 2.5.2. Mapping ALOS

ALOS was subset to approximately 41 km^2^, or 3.7% of the whole 1,000 km^2^, at the west side of the study area. Prior to that, ALOS land cover types were derived from unsupervised classification. First the image had been classified into five land covers and were reclassified into four types because we are interested in assessing accuracy for the massive pixel size of MODIS, though, only the open areas, forests, oil palm and rubber plantations were considered in clustering; the others were merged and grouped as unclassified. Overall techniques employed to derive final map of ALOS incorporated of reclassification, proximity analysis and spatial analyst of major filtering by using ARC GIS 10.0 as reported in Table 3.

### 2.6. Sampling Points and Accuracy Assessment of MODIS Land Use Classification

We used ground verification survey, NFI-4 and Topographic data 1997, MOD12Q1, ALOS land cover type and elevation to evaluate the accuracy of the MODIS land use classification, since accuracy assessment is a critical step in analysing any map created from remotely sensed data [21]. Standard assessment of accuracy included Producer's, User's and Overall Accuracy were employed for accuracy assessment [31–33]. The accuracy data were derived from error matrices table to find the reliability and accuracy of the maps produced [34]. The accuracy is a direct interpretation of percentage of cases correctly classified [35]. Producer's Accuracy indicates the probability of a reference pixel being correctly classified. User's Accuracy is where if the total number of corrected pixels in a category is divided by the total number of pixels that were classified in the category [36]. Overall Accuracy is the simplest and one of the most popular accuracy measures computed by dividing the total account (*i.e.*, the total sum of the major diagonal) by the total amount of pixels in the error matrix [31].

#### 2.6.1. Comparison with NFI-4 and Topographic Data 1997

We employed stratified random sampling points in order to assess the accuracy for MODIS land use classification. Because of low resolution of the MODIS satellite image employed and inaccessibly of the forested areas except for the central point (PFR) areas and agricultural areas limited number of sampling points were qualifying to locate and survey. This is because a low number of points may contribute to errors [37]. Therefore, to supplement this, we used NFI-4 data to input more points which generated a total of 4,791 points on the MODIS land use classification. The points generated were for four different categories such as forests, oil palm, rubber, and mixed horticulture. We used the NFI-4 data because it was produced for long-term Malaysian forest inventory resources database (2000–2010), which also incorporated SPOT image of 2010 for delineation of forested area. Furthermore, Topographic data 1997 (sheet codes 3957b, 3957d, 4056a and 4057c in CAD format); which had been ground proofed by the Malaysian Survey and Mapping Department was used for generation of sampling points. The complete data employed was presented in Table 4.

**Figure 3.**
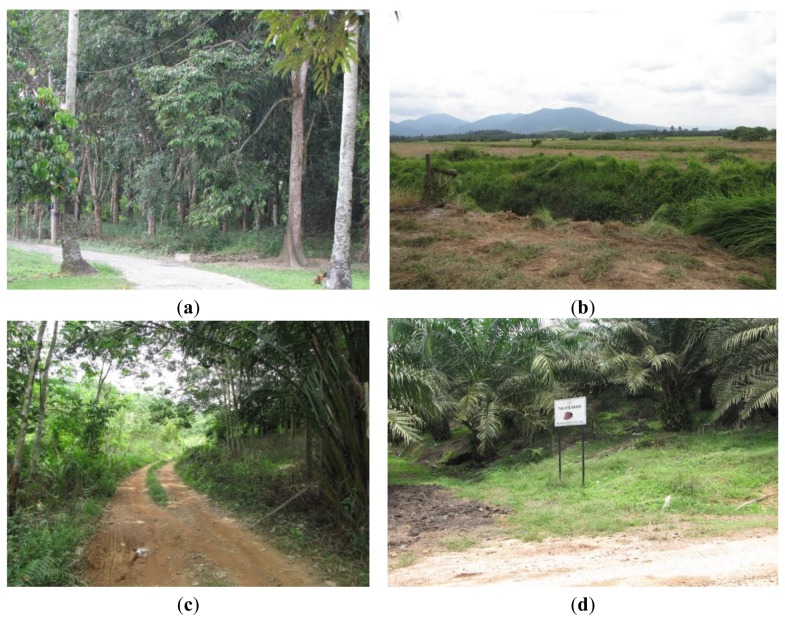
Ground proofing photos for the study area. (**a**) A housing area in the rubber and oil palm estate which also contained langsat (*Langsium domesticum*) trees, mangosteen (*Garcinia mangostana*) and rubber; (**b**) Abandoned paddy field; (**c**) Rubber trees; (**d**) Oil Palm trees.

Subsequently, all the points were ground verified to obtain an error matrix and overall accuracy of the classification. The areas surveyed included oil palm and rubber plantations, forests areas, paddy fields, and housing areas located among crop trees such as langsat (*Langsium domesticum*) trees, mangosteen (*Garcinia mangostana*) and coconut trees (Figure 3). The survey was started on 24 October 2011 and ended at the end of March 2012 with Global Positioning System (GPS) and digital camera as the main information capture tools. In order to conduct further comparison, percentage of land use classes were also derived.

#### 2.6.2. Comparison with MOD12Q1

**Figure 4.**
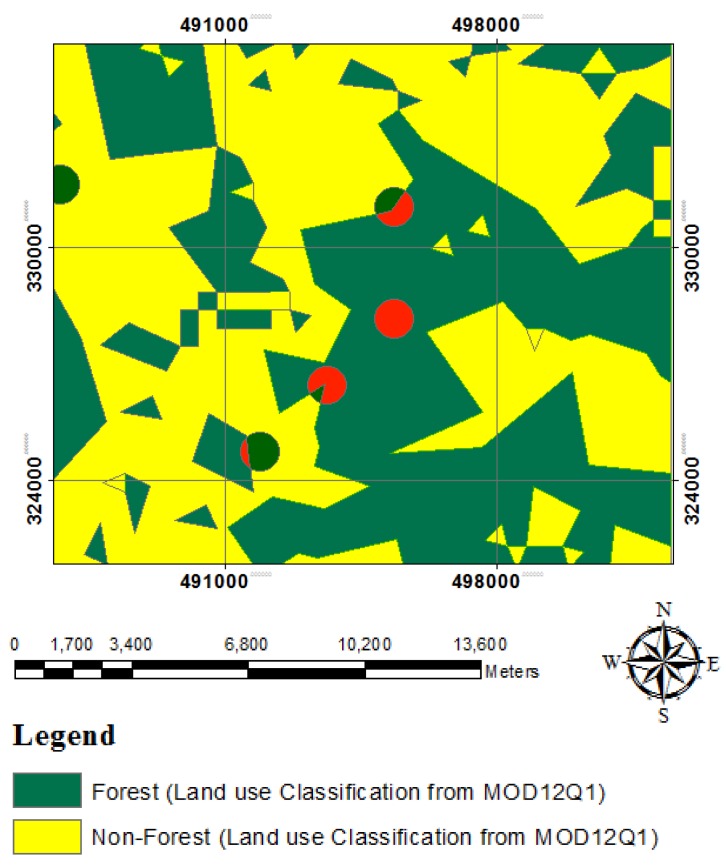
Land use classification sample points from MOD09A1 (red circle), which are covered by at least 95% pure on MOD12Q1 were assigned to the dominant cover (“forest or non-forest”)-red colour, while points of land use classification sample points from MOD09A1 that were below 95% were assigned as (“forest or non-forest”) class.

This study extracted three data sets: MODIS 2000 ISODATA-2, MODIS 2005 K-Means-1 and MODIS 2008 ISODATA-1 for further development of accuracy assessment with MOD12Q1 data sets as a result of a successful classification of those pixels into a land use classification. The land use classification of MODIS 2000, 2005 and 2008 have overall accuracy of 85%, 65% and 94%, respectively. A comparison between the land use classification and MOD12Q1 for all data sets was conducted, and an error matrix was generated to evaluate the consistency of the land cover classification results [4]. MOD12Q1 data sets were regrouped into forest and non-forest based on NFI-4 data. MOD12Q1 evergreen broadleaf forest is regrouped into forest and others as non-forest category. In this study, sample points of land use classification from MOD09A1 which covered as at least 95% pure on MOD12Q1 were assigned to the dominant cover (“forest or non-forest”), while points of our land use classification from MOD09A1 that were below 95% on MOD12Q1 were assigned as (“forest or non-forest”) class (Figure 4). Previously, the sampling points on MOD12Q1 were buffered at 500 m, extracted and overlaid on the MODIS land use classification. The objective was to link with MOD12Q1 data to improve the purity level of the classification and to assess accuracy as modified by [33] such as sites that were at least 70% pure were assigned to the dominant cover type, while mixed sites (e.g., 67% conifer and 35% herbaceous) were classified as mixed coniferous/herbaceous. The objective of the appointment of purity was to avoid confusion during the evaluation of an accuracy of the points reaching the designated threshold.

#### 2.6.3. Validation of ALOS Land Cover Types

In addition, sample images from Google Earth in 2008 were used as reference to the ALOS land cover types accuracy assessment. We matched and validated rubber and urban areas with Google Earth images of Thailand which were the areas studied by [38,39]. An area from a non-traditional rubber plantation planted on 10,000 ha to 50,000 ha in Kuan Wan, Thailand which is near the border of Cambodia [38] was used in the study. In addition, rubber estates in Kemayan, Negeri Sembilan and an open area in several areas in Penang Island of the peninsula were also incorporated in the study. Land Surface Temperature (LST) product derived from Landsat TM in a study conducted by [39] were used to compare with urban areas, since, LST measure temperatures from land surface. The surface temperature (T_s_) is related to percentage of green cover, hence, the lower the green cover the higher the surface temperature.

#### 2.6.4. Comparison with ALOS Land Covers Type

Once a classification map is developed in this way, it needs to be validated against known data. Researchers have been validating their maps with available global satellite data land cover products such as the MODIS Land Cover Type product (MLC) [40,41]; Landsat-based National Land Cover datasets—for example, the IKONOS-derived forest map [42], China's database (NLCD) [43] and Google Earth [38], which has a high horizontal potential accuracy [44]. Mapping forests with ALOS PALSAR 50-m data, for example, was successfully used to differentiate between primary forest and newly deforested areas in the Brazilian Amazon [45]. However, we might have needed more ALOS data to represent our area, which significantly increased time and cost. Therefore, a combination of MODIS land use classification and highly satellite resolution data was the most feasible method of land use mapping in our tropical forest. The MODIS land use classification of 2008 (highest overall accuracy) was overlaid to compared and assess spatial distribution of the land use classification on higher resolution satellite image as a sample from all the maps.

#### 2.6.5. Elevation Map

The elevation map was derived from following standard geo-statistical procedure of kriging interpolation analysis conducted in the ARC GIS 10.0. Elevation play a huge role in differentiating in soil and light resources hence appears related to stature of the forest [46]. A relevant study by [47] on species richness of different elevational of Mount Kinabalu (Borneo) tropical rainforests found species pool among forests was one of the causal interpretation among dynamics, productivity and species richness of the study. Study [48] has revealed rainforests from lower slopes up to 300 m elevation comprise the mixed Dipterocarpus community. In this sense, we sought to examine the distribution of forest clusters, again with the land use classification of MODIS 2008 (ISODATA-1) (highest accuracy) with elevation as a sample. In addition, the NFI-4 data was overlaid with elevation to further evaluate and validated the spatial distribution of land use classification (only for forest class).

## 3. Results and Discussion

### 3.1. MODIS Land Use Classification

Overall classification methods within an overall accuracy of 57% to 94% and percentage of the clusters area are given in Table 5 and the results of the accuracies were depicted in Table 6. As seen in Table 6 Forest was classified in all the data set maps excluding those for ISODATA-1 from MODIS 2005.

The unclassified Forest from MODIS 2005 shows that forest areas in the data were underestimated in the southern part of the study area, as thin clouds over the forest were misclassified as crops. In Table 6 the Producer's Accuracy for the Forest was highest in data sets from MODIS 2000 for K-Means-2 (87%) and lowest in data sets MODIS 2005 (71%) for K-Means-1. User's Accuracy for the Forest was highest in data set MODIS 2000 and MODIS 2005 for K-Means-2 and K-Means-1 (100%). User's Accuracy for the Forest was lowest in data set MODIS 2008 (90%) for both ISODATA-1 and ISODATA-2. Table 6 also depicted the highest overall accuracy was 94% for the data set MODIS 2008 for ISODATA-1, while the lowest overall accuracy was 57% for MODIS 2005, ISODATA-1. MODIS 2005 land use map had lower accuracy than 2000 because the image consisted with thin cloud cover over the forest areas. This is because tropical forest areas are a difficult site to obtain good quality satellite data due to heavy cloud cover conditions [22]. Although, the image was improved by atmospheric correction and cloud screening by MODIS science team [49] the image still influenced by minor cloud contamination.

Oil palm had the highest Producer's and User's Accuracy of 80% and 94%, respectively, in data sets from MODIS 2008 for ISODATA-1, where components of oil palm were also detected in every dataset map. However, in Table 6 Oil palm was misclassified as Mixed Oil palm and Rubber in datasets from MODIS 2000 for ISODATA-2 and in the datasets map from MODIS 2005 for ISODATA-1. Species such as *Calopogonium mucunoides, C. caeruleum, Centrosema pubescens* and *Pueraria phaseoloides* are legumes used as cover crops for oil palms for soil erosion control during the 8–10 months of land clearing [50]. In general, oil palm showed strong performance for accuracies in both data sets from MODIS 2008 for ISODATA-1 and ISODATA-2.

**Figure 5.**
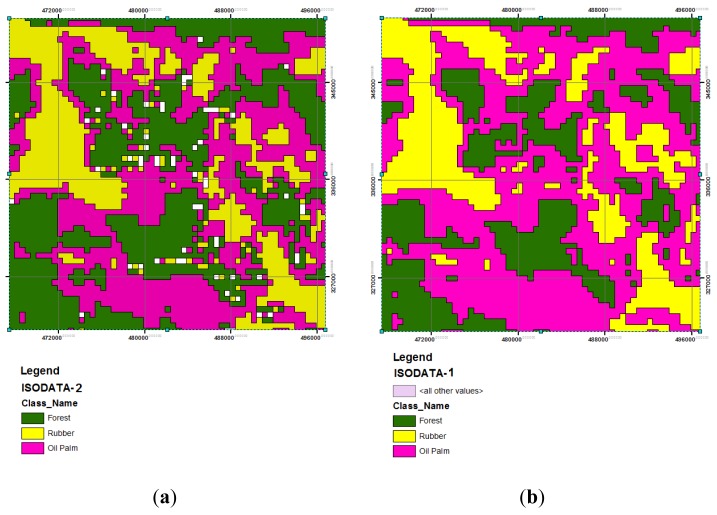
An example of land use classification of MODIS 2008. (**a**) ISODATA-2 (overall accuracy = 76%); (**b**) ISODATA-1 method (overall accuracy = 94%).

Heterogeneity of evergreen tropical forests was not acknowledged among the MODIS 2000 and 2005 dataset maps as reported in the results; however, this was relevant to MODIS 2008 for ISODATA-1 and ISODATA-2. An example of MODIS 2008 employing ISODATA-1 and ISODATA-2 is given in Figure 5. In general, Forest performed highly, as highlighted by a User's Accuracy of 90% and Producer's Accuracy of 86% from the MODIS 2008 dataset for ISODATA-2.

In this study, however, homogeneity of rubber trees (Rubber) in the plantation was misclassified into the more dominant evergreen forest. Multispectral reflectance of the trees leading to the misclassification led to over-estimation of the rubber area [38,51].

Overall, ISODATA and Gamma (with filtering window 5 × 5) classification were very successful at classifying MODIS pixels into forest and non-forest, although the MODIS 2005 data showed low overall accuracy and Forest percentage and also completely failed to discriminate the forest classification in ISODATA-1. Estimations of area percentage of MODIS land use classification for data sets were different among the ISODATA, K-Means methods, and datasets. The areas of land use classification are: 87% forest; 10% mixed oil palm and rubber; 3% mixed horticulture (MODIS 2000) (Table 5). The estimation of 10% incorporated mixed land uses indicated insufficient components or character of MODIS pixels to be classified into oil palm or rubber crops. The areas for MODIS 2005 were: 2% forest; 57% mixed forest and rubber; 39% oil palm; 2% mixed horticulture, indicating overlapping or misclassification of forest and rubber. The areas for MODIS 2008 were: 44% forest; 23% oil palm; 33% rubber, giving a better representation of the whole study area.

Finally, the study found ISODATA revealed its capability at classifying heterogeneous areas although overlapping occurred in MODIS 2005 ISODATA (forest with mixed oil palm and rubber classes). Again, misclassification may have been caused by thin or small areas of, cloud cover, which occurred in some places in the study area. Generally, most clouds occurred in tropical forests with frequent rainfall during the time the images were sensed. A study on the Bukit Soeharto evergreen tropical forest on the east coast of Kalimantan (Indonesia) had similar problems in obtaining good-quality satellite data due to a lower seasonality and heavy cloud-cover conditions [22,42,52]. We also highlighted that the loss of a large portion of forest classification in the MODIS 2005 for ISODATA-1 was not due to deforestation or human physical contact, but was a result of misclassification caused by the persistence of clouds in the image. ISODATA alone achieved 85%–94% overall accuracy, indicating that ISODATA classification was successful at classifying coarse-resolution pixels such as MODIS images. Finally, we found that the overall accuracy of the 2008 data sets ISODATA-1 was more than acceptable as compared to the control data and presented as the best land use classification in the study.

To explore the potential of MODIS image in the study, we found that an assortment of multi-temporal data effectively contributed to higher overall accuracy in the study. The MODIS 2000, MODIS 2005 and MODIS 2008 data represented a phenology of rubber in the study area, and since rubber is sensitive to temperature change, it has different phonological characteristics [4,22]. The MODIS Enhance Vegetation Index (EVI) satellite phenology map was depicted for vegetation activity in [22]. However, we found the map too coarse to be spotted and compared with our study map. Our desire in employing the data in the study is to present a more understandable MODIS capability in the classification of land use in tropical forest regions.

### 3.2. Validation of ALOS Land Covers Type with Google Earth

**Figure 6.**
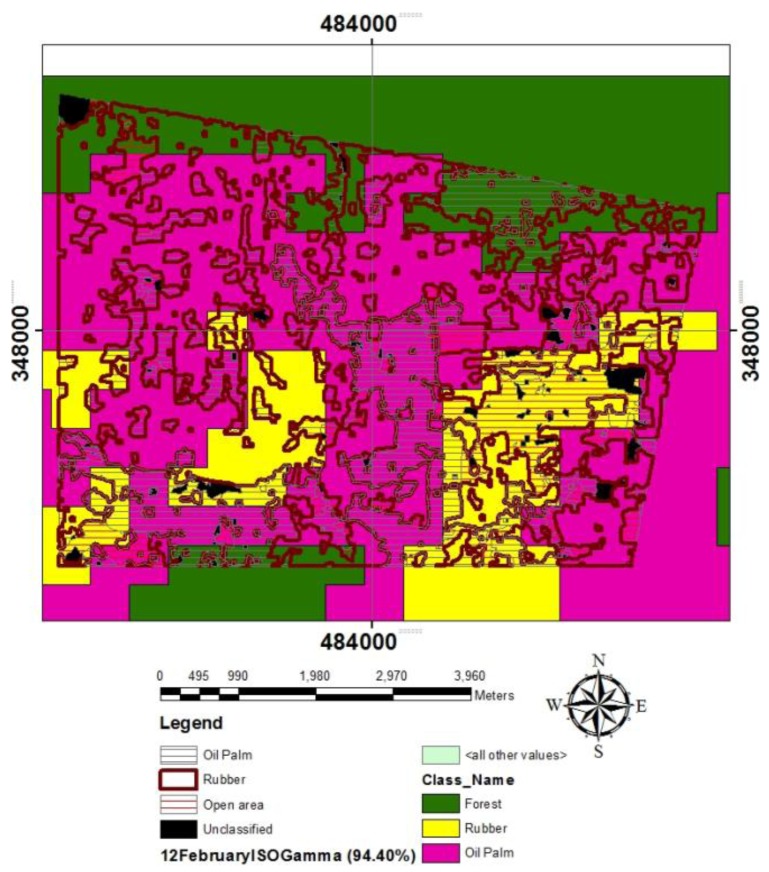
ALOS map overlaid with MODIS 2008 land use classification.

ALOS produced five land cover types, namely Open areas, Forests, Oil Palm, Rubber and Unclassified land cover. The map produced a 4.6 × 10^3^ km^2^ Open area; 2.3 × 10^4^ km^2^ Rubber; 1.27 × 104 km^2^; and a 1.04 km^2^ Unclassified area, which Forest was not included in the sampling area (3.7% of the whole 1,000 km^2^, at the west side) (Figure 6). Oil palm areas were also consistent with Google Earth which showed a comparable oil palm plantation adjacent to Kemayan, Negeri Sembilan. Open area which was identified to the same extent of higher reflectance after comparison with an area in Batu Maung, Bayan Lepas, Air Itam, George Town and Gelugor, in Penang Island at 100°15′E 5°20′N. The study found that both open areas and urban areas had higher reflectance, indicated by optimum Land Surface Temperature (LST) image in a study conducted by [39]. The results showed good agreement of ALOS with Google Earth that confirmed the capability of ALOS image to further compare with MODIS map.

### 3.3. Comparison of MODIS Land Use Classification and ALOS Map

Clusters of MODIS 2008 data such as Forest, Rubber and Oil Palm overlaid on the ALOS map showed various proportions (Figure 6). MODIS 2008 consisted with ALOS land cover types. Visually, young oil palm groves could be distinguished from open areas or rubber on the ALOS, whereas rubber was more easily distinguished from an open area than from mixed rubber and forest. Mapping MODIS land use, combined with unsupervised classification of low and higher satellite resolution, compromise a low-cost land-use mapping production process, and the data analysis can be rapidly performed.

### 3.4. Comparison of MODIS Land Use Classification and MOD12Q1

The accuracy results are given in Tables 7 and 8. The overall accuracy for purity > 95%, was 92%, which was the highest in the MODIS 2005 K-Means (K-Means-1). Overall, the highest Producer's Accuracy for forests was 73%; however, it was higher in User's Accuracy, which indicated higher success for user interpretation (100%) for 2000 and 2005 data. The Producer's Accuracy was 100% (MODIS 2000 and 2005) for non-forest, which separated the area very well and was expected to derive a higher User's Accuracy, of more than 80%; however it was low again in 2008. The regrouping of MOD12Q1 led to a higher Producer's Accuracy in the non-forest components.

Low overall accuracy for purity <95% averaged 55%. Forest had low Producer's Accuracies from MODIS 2000, MODIS 2005 and MODIS 2008 of 60%, 44%, and 24%, respectively. However, it achieved a good agreement with User's Accuracy for data sets from MODIS 2005 (90%), which again showed that the MOD12Q1 2005 was a very high-quality global land cover map derived from MODIS satellite imagery, which also showed in accuracy for purity of >95%.

As expected, visual interpretation of the comparison found that the PFR polygon delineated a good shape of forest reserved in the MOD12Q1 data, where most the sampling points for forest were distributed as observed in the map. This indicated a good agreement between the two products [33]. Visually, we also found that MOD12Q1 2001 and 2008 data sets inadequately presented at least the homogeneity of oil palm or rubber in the study area. We had limited ability to identify the age of the oil palm and rubber trees from our MODIS land use classification, as our sampling points were not located according to different ages of the trees. Thus, we expect some misclassification of rubber plantations and forests, due to the heterogeneity of forest trees and mixed land cover such as bushes and scrub within rubber trees. The survey found that the rubber trees were mature, but that the land was also occupied by fallow vegetation.

### 3.5. Comparison of MODIS Land Use Classifications with Elevation

**Figure 7.**
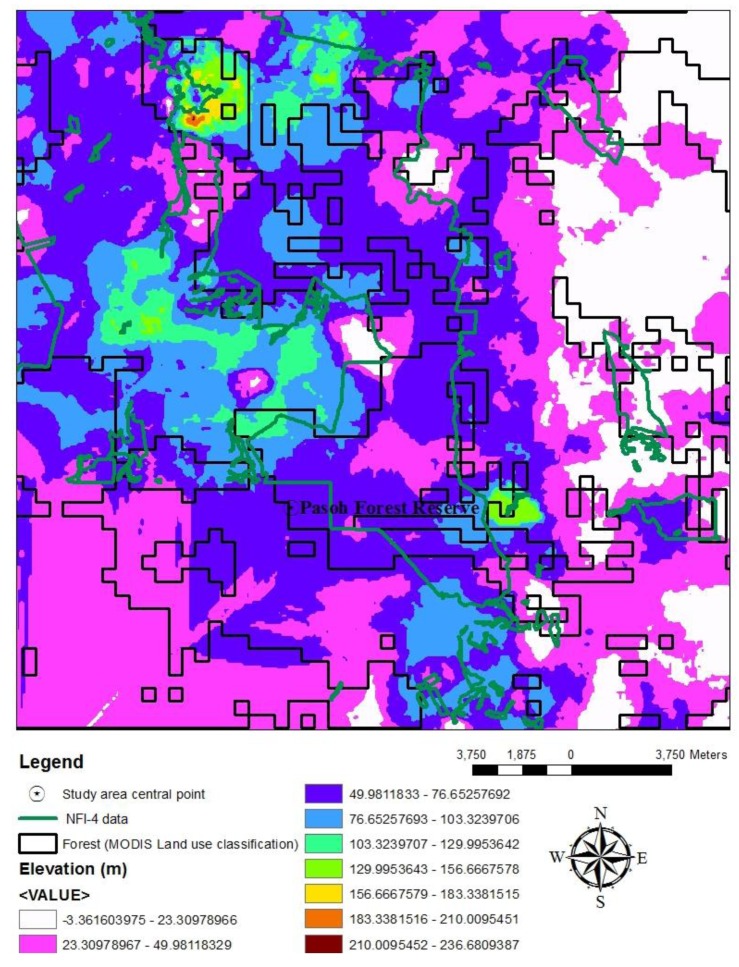
ALOS map overlaid with MODIS 2008 land use classification and NFI-4 data.

The elevation ranged from approximately 23 m to 236 m is overlaid with MODIS 2008 land use classification (Figure 7). The map showed the forests are concentrated mainly in the higher and moderate elevations, with 49–76 m at the highest levels, although PFR is located at a lower elevation: 75–103 m, which is consistent to a study by [12] and slightly agreed with [46]. The study found significant benefits in applying elevation to the land use classification, hence, enhanced better understanding of mixed Dipterocarpaceae species distribution at different elevation. It also had a good agreement with the data sets map for MODIS 2008 ISODATA-1 for forest clusters. Oil Palm data of MODIS 2008 was mainly distributed at a level similar to Forest: 103 m to 129 m, and Rubber was distributed much lower, at 23–49 m. Moreover, NFI-4 of forest class was observed to be consisted with the elevation, with distributed of mixed Dipterocarpaceae at the highest elevation in the study area.

## 4. Conclusions

This study evaluated the application potential of ISODATA and K-Means (Gamma and Kuan) classification for delineation and land use mapping of evergreen tropical forests, oil palm and rubber plantations, and other land uses in tropical zones. The study first constructed the accuracy assessment from our sampling methods. The most successful maps, ranging from 65% to 94% of overall accuracy, were then extracted for further comparisons. MODIS Land Cover map from the years of 2001, 2005 and 2008 were employed for accuracy assessment with the MODIS land use classification. Finally, we overlaid and compared the maps with NFI-4 data, Topographic data 1997, ALOS land cover type and elevation. The study revealed the advantages of using unsupervised ISODATA classification. This study recommends that future works be concentrated on matching regional or local vegetation densities information (surveys) to compare with the vegetation density from MODIS satellite data such as EVI and Normalized Difference Vegetation Index (NDVI) [9,18,22]. Generally, NDVI have different values in evergreen tropical forest, both young and mature rubber plantings, and open areas in young oil palm plantations [53]. By taking into account the vegetation indices, the map can be enhanced to show conditions as recent as the past 8 days, which can then be analysed for environmental stresses such as soil moisture stress, and can also be used for forest fire risk assessment [54], as it can even assist in distinguishing fuel types. For example, *Imperata* grassland present in an oil palm growing region is a flammable material, and has a higher combustion rate that can express the proportion of biomass likely to be consumed by fire [55].

The oil palm classification in this land use classification map is also valuable for providing information about natural pasture in the area, and which vegetation can be utilized for forage for livestock production [50], especially since the areas between the rows in young oil palm groves are usually covered with vegetation comprising legumes, grasses, broadleaf species and ferns. Consequently we also suggest that oil palm, which is classified by employing low-resolution imagery, should be recognized as mixed oil palm and other vegetation. We also recommend the [22] phenology vegetation activity map as a good foundation for phenology reference for future study in tropical forest land use classifications.

Sustainable Forest Management (SFM) is a dynamic and evolving concept aimed to maintain and enhance economic, social and environmental value of all type of forests for the benefits of present and future generations [25]. Robust economic development will remain in the medium-term as reported in 2013 particularly in Southeast Asia (surrounded by tropical evergreen broadleaf forest) [56], leaved those countries facing upcoming limited or cutting down expending allocation to certain governmental sectors. With constraints allocation of funds from government and private sectors to achieve the aims, SFM would be not meaningful. MODIS imaging showed capability to provide economically viable updated imageries and integrated land use mapping. MODIS imaging with integrated land use mapping, highlighted by using higher resolution of ALOS imagery, could assist forest managers to achieve SFM aims through increased frequency of land use mapping within the management areas with minimum labour and equipment cost. This is because MODIS enables deriving data at no cost, requiring a very low human labour cost with additional powerful computers. Moreover, Geographical Information System (GIS), land use mapping or remote sensing and GIS application unit at Forestry Departments could enhance their work through sustaining and updating their land use maps as a database which can be used by other governmental sectors.

With this study, we hope that an exploration of the development of land use maps for tropical forests will continue and will increase the usefulness of EO data in the future. This study revealed that there is insufficient information for a crop data base for the study area and for the peninsula as a whole, a situation that might be corrected with the application of MODIS imaging. For example, site suitability, soil suitability class, and agro-climatic region maps produced by the Malaysian Agricultural Department, do not include information on crop growth. But with a frequent data collection cycle (1–2 days) in 36 spectral imaging, maps could be produced for input and update to such a database. Furthermore, more rapid processing and analysis from higher-resolution remote sensing could lower the cost for image pre-processing.

## Figures and Tables

**Table 1.** Data used in the study.

**No.**	**Data**	**Resolution (meter)**	**Year**
1.	MODIS Surface Reflectance series data (MOD09A1)	500 m	2000, 2005, 2008
2.	MODIS Land Cover products (MOD12Q1) [19]	500 m	2001, 2005, 2008
3.	ALOS (Advanced Land Observing Satellite of Advanced Visible)	10 m	2008

**Table 2.** Description of the classification label assigned.

**Methodology**	**Description of Filtering (5 × 5) pixels**
ISODATA Gamma	ISODATA-1
ISODATA Kuan	ISODATA-2
K-Means Gamma	K-Means-1
K-Means Kuan	K-Means-2

**Table 3.** ALOS land cover types development techniques.

**Methodology**	**Parameters**
Reclassification	Natural Breaks

Proximity analysis	Buffering features at 500 m

Spatial Analyst with Majority Filter	Aggregate Cell Factor is “10–20”
	Boundary Clean is “Ascending”
	Number of Neighbours to use is “8”
	Replacement threshold is “Half”

**Table 4.** Data employed in the study for an accuracy assessment.

**Map**	**Scale**	**Source**	**Produced/Published, Year**
Present Land use—Negeri Sembilan 1997 and 2004	1:150,000	Malaysian Agricultural Department, Putrajaya	Malaysian Agricultural Department, Putrajaya/Soil Resource Conservation and Management Division, Malaysian Agricultural Department, 1997 and 2004
Topographic map—Seremban 1996 (Sheet 3856)	1:50,000	Universiti Putra Malaysia	JUPEM/Director of National Mapping, 1996
Topographic map—Kuala Pilah (Sheet 3956)			
Topographic Sheet Code (3957b, 3957d, 4056a, 4057c) (CAD format)	1:250,000		JUPEM/Director of National Mapping
NFI-4—2000–2010	Information not available	Peninsular Malaysian Forestry Department	Peninsular Malaysian Forestry Department, 2000–2010

Note: JUPEM (Malaysian Survey and Mapping Department).

**Table 5.** Overall land use/land cover produced with 57%–94% overall accuracy.

**MODIS Land use classification Map**	**Classification with Overall Accuracy (57%–94%)**	**Land Use/Land Cover Classes, Area (%)**
MODIS 2000	ISODATA-2	Forest (87), Mixed Oil Palm and Rubber (10); Mixed Horticulture (3)
	K-Means-2	Forest (2), Mixed Oil Palm Rubber Oil Palm (79), Mixed Horticulture (19)
MODIS 2005	ISODATA-1	Mixed Oil palm and Rubber (12); Oil Palm (76), Mixed Horticulture (12)
	K-Means-1	Forest (4), Mixed Forest and Rubber (57); Oil Palm, Mixed Horticulture (39)
MODIS 2008	ISODATA-1	Forest (44), Oil Palm (23), Rubber (33)
	ISODATA-2	Forest (39), Oil Palm (16), Rubber (45)
ALOS	Reclassified, Proximity analysis and Spatial Analyst	Open areas, Forests, Oil Palm, Rubber, Unclassified (Area not tested)

**Table 6.** Data employed in the study for an accuracy assessment.

**Land Use Map**	**Classification**	**Land use types**	**Producer's Accuracy**	**User's Accuracy**	**Overall Accuracy**
	ISODATA-2	Forest	60	90	85
		Mixed Oil Palm and Rubber	53	53	
		Mixed Horticulture	0	33	
MODIS 2000	K-Means-2	Forest	87	100	67
		Mixed Oil Palm and Rubber	53	45	
		Oil Palm	50	50	
MODIS 2005	ISODATA-1	Mixed Oil Palm and Rubber	55	50	57
		Oil Palm	50	67	
		Mixed Horticulture	50	33	
	K-Means-1	Forest	71	100	65
		Mixed Forest and Rubber	67	74	
		Oil Palm	63	83	
		Mixed Horticulture	63	33	
MODIS 2008	ISODATA-1	Forest	78	90	94
		Oil Palm	80	94	
		Rubber	45	29	
	ISODATA-2	Forest	86	90	76
		Oil Palm	76	94	
		Rubber	67	47	

**Table 7.** An accuracy assessment between land use classification and MOD12Q1data sets (sampling points >95% purity).

MODIS 2000 ISODATA-2	**Forest**	**Non-Forest**	**Total**	**UA (%)**
Forest	20	0	20	100
Non-Forest	8	33	41	80
Total	28	33	61	
PA (%)	71	100		87

MODIS 2005 K-Means-1	Forest	Non-Forest	Total	UA (%)

Forest	20	0	20	100
Non-Forest	8	36	41	88
Total	28	36	61	
PA (%)	71	100		92

MODIS 2008 ISODATA-1	Forest	Non-Forest	Total	UA (%)

Forest	11	9	20	55
Non-Forest	4	37	41	61
Total	15	46	61	
PA (%)	73	80		79

Note: PA = Producer's Accuracy; UA = User's Accuracy.

**Table 8.** An accuracy assessment between land use classification and MOD12Q1 data sets (sampling points <95% purity).

MODIS 2000 ISODATA-2	**Forest**	**Non-Forest**	**Total**	**UA (%)**
Forest	12	8	20	60
Non-Forest	20	21	41	51
Total	32	29	61	
PA (%)	60	72		54

MODIS 2005 K-Means-1	Forest	Non-Forest	Total	UA (%)

Forest	18	2	20	90
Non-Forest	23	18	41	44
Total	41	20	61	
PA (%)	44	90		59

MODIS 2008 ISODATA-1	Forest	Non-Forest	Total	UA (%)

Forest	4	16	20	20
Non-Forest	13	24	41	68
Total	17	44	61	
PA (%)	24	64		52

Note: PA = Producer's Accuracy; UA = User's Accuracy.
